# Assembly and Entry of Severe Acute Respiratory Syndrome Coronavirus 2 (SARS-CoV2): Evaluation Using Virus-Like Particles

**DOI:** 10.3390/cells10040853

**Published:** 2021-04-09

**Authors:** Binod Kumar, Grant M. Hawkins, Tom Kicmal, Enya Qing, Emily Timm, Tom Gallagher

**Affiliations:** Department of Microbiology and Immunology, Loyola University Chicago, Maywood, IL 60153, USA; bkumar@luc.edu (B.K.); ghawkins1@luc.edu (G.M.H.); tkicmal@luc.edu (T.K.); eqing1@luc.edu (E.Q.); etimm1@luc.edu (E.T.)

**Keywords:** coronavirus, SARS-CoV2, D614G, MERS-CoV, MHV, virus-like particles, virus assembly, nucleocapsid, release, COVID-19, HiBiT, LgBiT, NanoBiT technology

## Abstract

Research on infectious severe acute respiratory syndrome coronavirus 2 (SARS-CoV2) is currently restricted to BSL-3 laboratories. SARS-CoV2 virus-like particles (VLPs) offer a BSL-1, replication-incompetent system that can be used to evaluate virus assembly and virus-cell entry processes in tractable cell culture conditions. Here, we describe a SARS-CoV2 VLP system that utilizes nanoluciferase (Nluc) fragment complementation to track assembly and entry. We utilized the system in two ways. Firstly, we investigated the requirements for VLP assembly. VLPs were produced by concomitant synthesis of three viral membrane proteins, spike (S), envelope (E), and matrix (M), along with the cytoplasmic nucleocapsid (N). We discovered that VLP production and secretion were highly dependent on N proteins. N proteins from related betacoronaviruses variably substituted for the homologous SARS-CoV2 N, and chimeric betacoronavirus N proteins effectively supported VLP production if they contained SARS-CoV2 N carboxy-terminal domains (CTD). This established the CTDs as critical features of virus particle assembly. Secondly, we utilized the system by investigating virus-cell entry. VLPs were produced with Nluc peptide fragments appended to E, M, or N proteins, with each subsequently inoculated into target cells expressing complementary Nluc fragments. Complementation into functional Nluc was used to assess virus-cell entry. We discovered that each of the VLPs were effective at monitoring virus-cell entry, to various extents, in ways that depended on host cell susceptibility factors. Overall, we have developed and utilized a VLP system that has proven useful in identifying SARS-CoV2 assembly and entry features.

## 1. Introduction

Coronaviruses (CoVs) include a remarkable diversity of animal and human-infecting species. There are seven human-infecting species, each causing disease of varying severity, ranging from mild or asymptomatic infections resembling the common cold to severe wheezing, bronchitis, or pneumonia [1,2]. These human CoVs are descendants of animal viruses that have invaded zoonotically [3]. There are a remarkable diversity of animal-infecting CoV species, and while most appear limited to their natural animal reservoirs, the emergence of severe acute respiratory syndrome coronavirus (SARS-CoV) in 2002 [4] and Middle East respiratory syndrome coronavirus (MERS-CoV) in 2012 [5], and SARS-CoV2 in 2019 [6], clearly demonstrated zoonotic CoV capabilities [3,7]. During December 2019, SARS-CoV2 was first reported in China, and within months, spread across the globe, causing pandemic coronavirus disease 2019 (COVID-19), with more than 2 million fatalities as of February 2021 [8].

Enveloped CoV particles enclose a single positive-sense RNA genome. The limiting envelopes contain three major proteins, matrix (M), envelope (E), and spike (S). M glycoproteins, which have three envelope-spanning transmembrane domains, are the most abundant CoV membrane proteins. The transmembrane domains are involved in M oligomerization, which generates a lattice-like network that is considered fundamental in driving CoV particle budding into the lumen of intracellular membranous organelles, principally the ER-Golgi intermediate compartment [9,10]. The coronavirus E protein is a minor but indispensable component of the virions [11]. The mechanisms by which E proteins operate in CoV assembly remains enigmatic, in part because E is abundantly expressed in infected cells but only very small amounts are incorporated into the virions, and because CoVs vary in their dependence on E for particle assembly and secretion [12,13]. The roles for E in assembly and secretion may be related to viroporin (ion channel) [14,15] and to membrane-deforming capacities that induce curvature at budding sites [16,17,18]. Several reports on mouse hepatitis virus (MHV), bovine coronavirus, and infectious bronchitis virus show that co-synthesis of M and E are sufficient for virus-like particle (VLP) assembly [19,20,21,22]. S glycoproteins are integrated into the lattice-like M networks and are essentially for virus-cell entry but are entirely dispensable in CoV assembly.

While several studies have documented that the membrane protein (M) and small envelope protein (E) are sufficient to form coronavirus VLPs, the question of whether these two proteins are all that is needed for efficient SARS-CoV2 assembly requires further investigation. Notably, the role of the cytoplasmic nucleocapsid protein (N) in virus assembly needs further investigations. N proteins bind virion genomic RNAs, forming helical ribonucleoprotein (RNP) complexes, which interact with M proteins and may in turn promote virus particle assembly [23]. N proteins are multidomain structures, with general architectures including amino terminal RNA binding domains (NTDs), serine- and arginine-rich (SR) linkers, and carboxy-terminal dimerization domains (CTDs) [24,25]. Domain-specific functions, however, appear to be variable. For avian infectious bronchitis virus (IBV-CoV), the NTDs bind viral RNA and the CTDs dimerize to generate higher-order RNPs [26], while for MHV-CoVs, the CTDs operate in recognizing viral RNAs [27]. These studies reveal knowledge gaps that justify further study of N proteins in the process of CoV particle assembly.

With the aim to further dissect CoV assembly processes, we developed a SARS-CoV2 VLP system that mimics the natural SARS-CoV2 assembly and cell entry processes. The VLP system utilizes an 11 amino acid Nluc fragment called “HiBiT”, which is appended to CoV structural proteins and serves as a marker for virus assembly, secretion from VLP-producing cells, and subsequent VLP entry into target cells. The HiBiT marker is identified by protein complementation with a larger Nluc fragment called “LgBiT”, with resulting Nluc detection by luminometry [28]. Here, we have used the HiBiT-VLP assembly system to delineate the crucial roles of SARS-CoV2 N protein in virus assembly. We generated chimeric betacoronavirus N proteins to identify the roles for specific N domains in VLP assembly. We also used the VLP system to track virus-cell entry, in ways that more faithfully represent authentic CoV-cell entry than other CoV pseudotype systems [29]. Overall, the VLP systems described here facilitate studies of virus assembly and entry in BSL-1 laboratory settings.

## 2. Materials and Methods

### 2.1. Cells and Culture Conditions

HeLa cells (ATCC) and human embryonic kidney cells (HEK-293T; ATCC) were grown in the complete Dulbecco’s Modified Eagle’s Medium (DMEM) supplemented with 10% (*v*/*v*) fetal bovine serum (FBS, Atlanta Biologicals, Minneapolis, MN, USA) and 1% penicillin-streptomycin (Gibco, Waltham, MA, USA). All the cells were cultured at 37 °C in a 5% CO_2_ incubator and used for experiments after only short-term passaging. All cell cultures were confirmed to be negative for mycoplasma as determined by myco PCR testing.

### 2.2. Reagents and Antibodies

Commercial reagents included nanoglo luciferase substrate and assay reagents (Promega Corporation, Madison, WI; USA), CMV LgBiT expression plasmid (Promega Corporation, USA), restriction enzymes (Thermo Fisher Scientific, Waltham, MA, USA), PCR and Gibson fragment assembly reagents (NEB), and size-exclusion columns (Izon Science Ltd., Medford, MA, USA). Rabbit polyclonal antibodies to detect the S (#JH5052), E (#JH5046), and M (#JH5054) of SARS-CoV2 were kind gift from Carolyn Machamer (Johns Hopkins University, USA).

### 2.3. Generation of Expression Plasmids

Human codon-optimized SARS-CoV2-S cDNAs were obtained from genscript (MC_0101081). Human codon optimized SARS-CoV2 E, M, N (GenBank: MN908947.3), and HiBit gene blocks were commercially synthesized (IDT, USA). All DNAs were cloned using Gibson assembly. The SARS-CoV2 E and M genes were cloned in pcDNA3.1+ mammalian expression vector. The SARS-CoV2 N protein was tagged with an 11 amino acid peptide tag (HiBiT). The HiBit-N gene was also cloned in a pcDNA3.1+ expression vector by ligating the HiBiT-linker sequence GTGAGCGGCTGGCGGCTGTTCAAGAAGATTAGCGGCTCGACCGGTGGCTCGAGCGGT, encoding the HiBiT tag (VSGWRLFKKIS) and sequence encoding a linker (GSTGSSG) to the 5′ end of the coding sequences for *N* gene (Figure 2A). Similarly, the SARS-CoV2 E-HiBiT and SARS-CoV2 M-HiBiT genes were cloned in same parent vector with the HiBiT tag at the 3′ end of E and M genes, respectively. MERS-CoV HiBiT-N, MHV HiBiT-N, as well as HiBiT-tagged SARS-CoV2 N-NTD (1–175) and N-CTD (247–419) were cloned into pcDNA3.1+ expression vectors. Chimeric-HiBiT-N constructs were cloned using Gibson assembly. Briefly, the various gene fragments (encoding different domains of N protein) of MERS-CoV (GenBank: JX869059.2) and SARS-CoV2 were selectively obtained by performing PCR, followed by cloning into pcDNA3.1+ expression vector. The Nluc-N was cloned by tagging the NanoLuc gene to the 5′ end of the coding sequences for *N* gene. All clones were validated by sequencing, and expression was measured using the Promega’s Binary NanoLuc Technology (NanoBiT) as per manufacturer’s instructions.

### 2.4. VLP Production and Purification

Adherent HEK293T cells at 80% confluence were co-transfected with equimolar amounts of plasmids encoding the CoV-S, E, M, and HiBiT-N proteins using LipoD transfection reagent (SignaGen, Frederick, MD, USA). 1:3 DNA:LipoD mixtures were mixed in serum free-DMEM for 10 min at room temperature followed by dropwise addition onto cells. Four h later, media were replenished with DMEM-1% FBS. Media containing VLPs were collected at 24 h post-transfection, clarified by sequential centrifugation, first at 300× *g*, 4 °C, 10 min, then at 3000× *g*, 4 °C, 10 min. VLPs in media were then concentrated 20-fold by ultrafiltration using 100 K Amicon ultra filters (MilliporeSigma, St. Louis, MO; USA), and 0.5-mL volumes were then applied to size-exclusion chromatography (SEC) columns. Fractions were collected according to manufacturer’s instructions (Izon Science Ltd., Medford, MA, USA), and VLPs were identified in eluted fractions using Nano-Glo luciferase assay reagents (Promega, Inc.). Briefly, aliqouts of each fraction were mixed with LgBiT protein in the presence of Nano-Glo luciferase assay substrate (Promega), and Nluc enzyme levels were measured in terms of relative light units (RLU) using a Veritas microplate luminometer. Fractions containing HiBiT-VLPs were aliquoted and stored at −80 °C.

### 2.5. Western Immunoblot Analysis

Cells were lysed using radioimmunoprecipitation assay (RIPA) lysis buffer (15 mM NaCl, 1 mM MgCl_2_, 1 mM MnCl_2_, 2 mM phenylmethylsulfonyl fluoride and protease inhibitor mixture [MilliporeSigma, USA]), lysates mixed 5:1 with 6× sample solubilizer (0.0625 M Tris·HCl (pH 6.8), 10% glycerol, 0.01% bromophenol blue, 2% (*w*/*v*) SDS, 2% 2-mercaptoethanol), and then heated at 95 °C for 5 min. Proteins were electrophoresed on discontinuous Laemmli polyacrylamide gels, transferred to nitrocellulose membranes (Bio-Rad, Hercules, CA, USA), and probed with primary antibodies and secondary HRP-conjugated antibodies and antibodies visualized by chemiluminescence (Thermo Fisher Scientific) as per manufacturer’s instructions. HiBiT-tagged proteins were identified by probing nitrocellulose membranes for 1 min with LgBiT protein in the presence of Nano-Glo luciferase assay substrate (Promega), followed by chemiluminescent visualization. Image processing was performed using FlourChem E (Protein Simple).

### 2.6. Binding and Entry Assay of SARS-CoV2 VLPs

For the binding assay, HeLa and HeLa-ACE2 cells were incubated with the normalized Nluc input multiplicities of EMN (E+M+N) and SEMN (S+E+M+N) containing VLPs for 1.5 h at 4 °C. The cells were washed and lysed to quantify the associated Nluc activity using the Promega’s Nano-Glo luciferase assay system. The EMN VLPs were used to calculate the background.

For the VLP-cell entry assay, HeLa cells were transfected with pcDNA-ACE2-LgBiT. Twenty-four h later, cells were inoculated with EMN and SEMN, containing VLPs for 1 h at 4 °C. Thereafter, cells were incubated with or without trypsin (20 ng/µL) in the presence of Nluc live cell substrate vivazine and transferred to 37 °C to initiate VLP-cell entry. Nluc activity was measured at various time points to assess kinetics of VLP entry. After 2 h, cells were dissolved in Nano Glo lysis buffer (Promega Corporation, Madison, WI, USA) to allow for maximal HiBiT:LgBiT complementation. The proportions of VLPs that entered cells in the 2 h time period were estimated relative to the maximal complementation after detergent cell lysis.

### 2.7. Statistical Analysis

Results are expressed as means ± SE of the mean of at least three independent experiments. Unpaired *t*-test and ANOVA with Dunnette post hoc test were applied to statistical differences amongst the groups. In all the experiments, *p* < 0.05 was considered statistically significant.

## 3. Results

### 3.1. Determinants of SARS-CoV2 VLP Assembly

**Figure 1:**
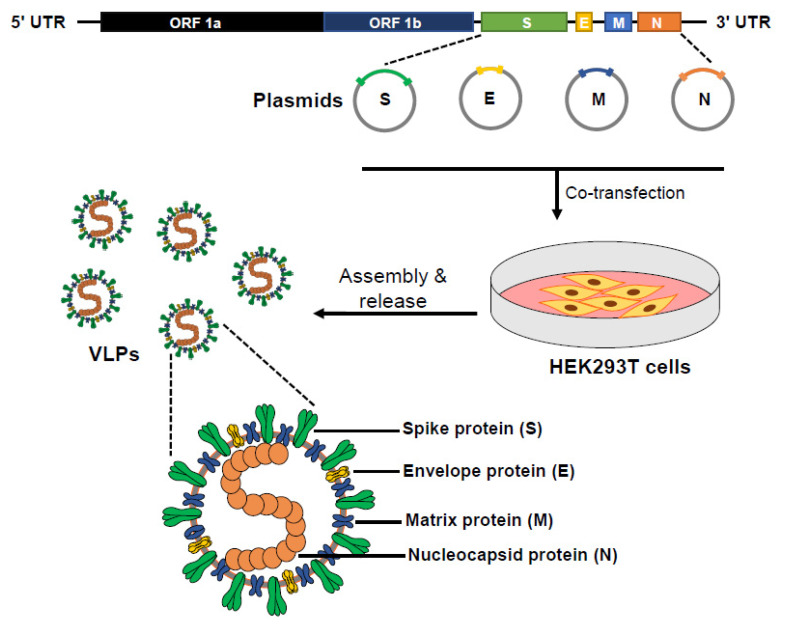
Schematic outline of SARS-CoV2 virus-like particle (VLP) production. Plasmids encoding severe acute respiratory syndrome coronavirus 2 (SARS-CoV2) structural proteins E, M, and N can be transfected in a suitable cell line, with or without plasmids encoding S proteins. VLPs are collected from culture media between 24 and 72 h post-transfection.

**Figure 2:**
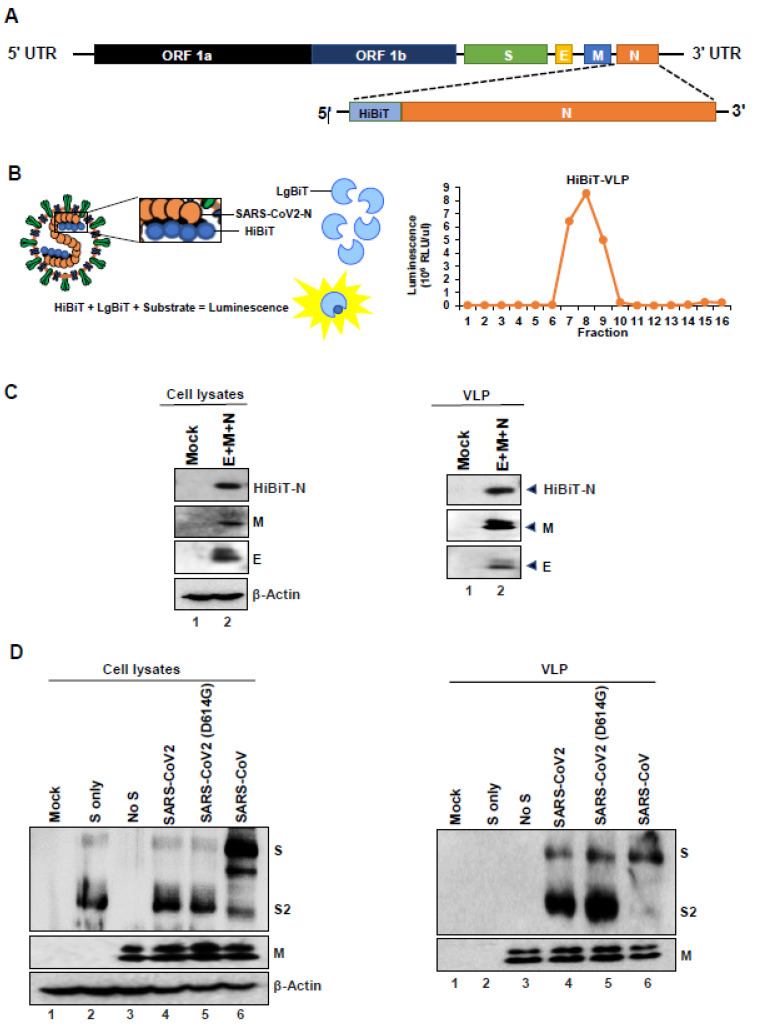
Production of SARS-CoV2 VLPs. (**A**) 11-amino acid HiBiT peptide was appended to the amino-terminus of N protein. (**B**) HiBiT-N within VLPs is detected using Binary NanoLuc Technology (NanoBiT). Addition of LgBiT protein and Nluc substrate generates luminescence within minutes of incubation. The right panel depicts elution of HiBiT-VLPs from size-exclusion chromatography (SEC) columns at or near void volumes (fractions 7–9). (**C**) Western blot (WB) images depict E, M, and N proteins in cell lysates of plasmid-transfected HEK-293T cells (left) and in size-exclusion chromatography (SEC)-purified VLPs (right). VLPs were harvested at 24 h post-transfection. (**D**) Plasmids encoding the spike protein of SARS-CoV2, SARS-CoV2 variant (D614G), and SARS-CoV were co-transfected with the E, M, and HiBiT-N of SARS-CoV2. WB images depict S and M proteins in cell lysates (left) and purified VLPs (right). SARS-CoV2 S proteins are cleaved into S1 and S2 during VLP secretion, while SARS-CoV S remains uncleaved.

CoV VLPs can be produced upon co-expression of the E, M, and N structural proteins in a mammalian expression system, and the S glycoprotein can efficiently incorporate onto these secreted VLPs [30]. Recent publications have also shown that SARS-CoV2 VLPs can be produced similarly upon co-expression of structural proteins. These studies have either used tagged M protein [31] or tagged E protein [32], both of which have been previously shown to be the minimal requirement for production of VLPs [33]. To determine optimal conditions for VLP production, we co-expressed the E, M, and N structural proteins in HEK-293T cells and collected the VLP-containing supernatant at early 24 h post-transfection times (Figure 1). We tagged the N protein with an 11 amino-acid peptide (HiBiT) for sensitive detection of secreted VLPs (Figure 2). We specifically chose to only tag the N protein because of our previous observation that revealed that the N protein of coronaviruses can tolerate amino-terminal extensions without affecting VLP production [34]. Secreted HiBiT-N VLPs were identified using Binary NanoLuc Technology (NanoBiT) (Figure 2B, left panel). Purified VLPs eluted from SEC columns at or near void volumes (fractions 7–9; Figure 2B, right panel). These SEC-purified VLPs contained E, M, and HiBiT-N, as evaluated by Western blotting (Figure 2C). S proteins were incorporated into VLPs when co-synthesized with E, M, and N (Figure 2D, right panel). Variant S proteins were equally incorporated when present (Figure 2D, right panel, lanes 5–6), making it clear that VLPs are suitable to evaluate S variants of concern. S proteins synthesized alone did not secrete (Figure 2D, right panel, lane 2).

### 3.2. Nucleocapsid Drives Efficient SARS-CoV2 VLP Production

**Figure 3:**
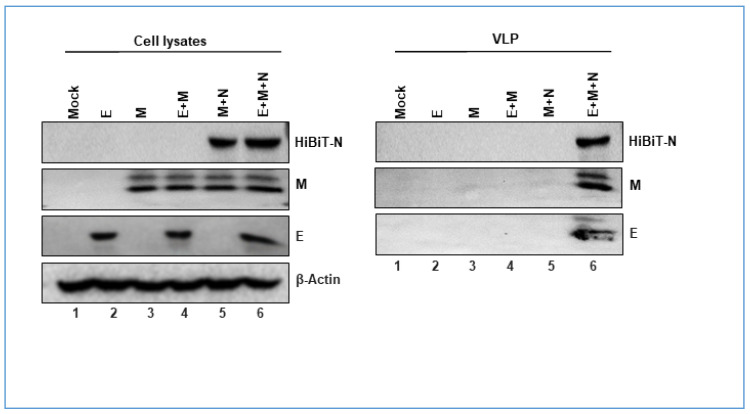
N proteins facilitate VLP production. The indicated plasmid co-transfections were performed, and 24 h later, cell lysates and SEC-purified VLPs were evaluated by WB for the presence of E, M, and N proteins.

To delineate the role of individual structural proteins in VLP assembly, we performed a series of transfections with E, M, and N, alone or together, purified secreted VLPs using SEC, and analyzed VLP proteins by Western blotting (Figure 3). Unlike previous reports showing that E and M were sufficient to produce VLPs, we identified dependence on E, M, and N proteins (Figure 3, right panel, lane 6). This finding fits more closely with a recent report demonstrating some dependence on N proteins for M secretion [32]. Of note, previous studies evaluated VLP secretion at relatively late times after structural gene expression (2 to 3 days post transfection), while we focused on earlier time frames, when structural proteins were less abundant and less likely to undergo unconventional secretion. Apparently, it is these conditions in which N proteins are central to VLP formation and secretion.

### 3.3. Nucleocapsid Carboxyl-Terminal Domains (CTDs) Drive Efficient VLP Production

**Figure 4:**
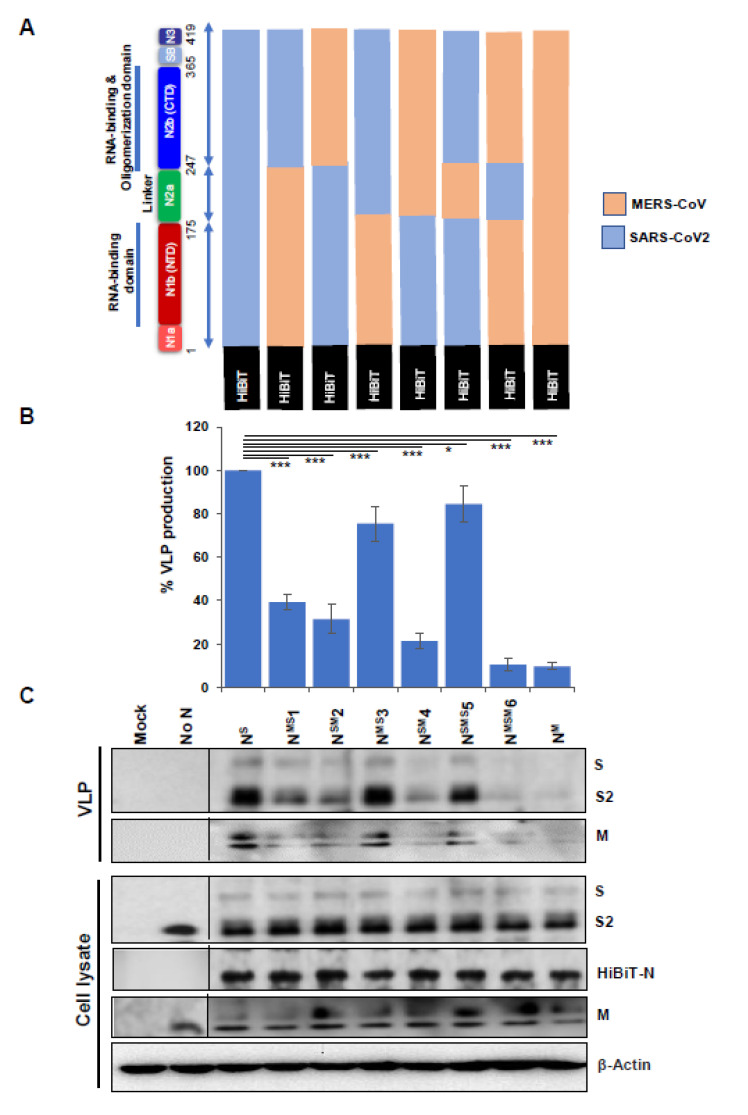
N protein CTDs facilitate VLP production. (**A**) Schematic diagram depicting SARS-CoV2/MERS-CoV N protein chimeras. Domains derived from SARS-CoV2 N proteins are in blue, with superscripts “S” and domains derived from MERS-CoV N proteins are in brown, with superscripts “M”. All chimeric N proteins are HiBiT-tagged. (**B**) HiBiT-N plasmids were co-transfected with SARS-CoV2 S, M, and E to produce VLPs. Yields of SEC-purified VLPs were determined by LgBiT complementation (NanoBiT technology). Results are means ±SE of three independent experiments. * *p* < 0.05, *** *p* < 0.001. (**C**) S, M, and N proteins in purified VLPs and cell lysates were detected by WB methods. Dotted lines separate blots obtained from separate experiments.

The central role for N proteins in VLP production (Figure 3) prompted us to ask whether individual N protein fragments might facilitate assembly. Towards this end, we constructed HiBiT-N NTD and CTD fragments to assess their support of VLP production. Unfortunately, these fragments accumulated at levels nearly 10 times lower than the full-length N protein (data not shown) and hence could not be utilized to obtain conclusive data. Therefore, we adopted an alternative approach in which we asked whether N proteins from related betacoronaviruses could replace SARS-CoV2 N. Betacoronavirus N proteins exhibit considerable variability; SARS-CoV2 N is 49% and 37% similar to MERS-CoV and MHV-CoV N, respectively. We expressed the MERS or MHV N proteins in conjunction with SARS-2 membrane proteins S, E, and M, and found that MERS N proteins were largely incapable of supporting assembly, providing only ~10% as many VLPs as the homologous SARS-2 N, as measured by tracking HiBiT-N secretion (Figure 4, panel B, rightmost lane). MHV N proteins were more capable in supporting SARS-2 VLPs, providing up to 40% VLP production relative to SARS-2 N (data not shown).

These differences between SARS-2 and MERS N proteins provided opportunities to delineate the roles of individual domains of N protein. We made a series of MERS-SARS-2 chimeric N constructs (Figure 4A). The chimeras divided the N protein into three major domains: (1) NTD; including the N1a + N1b domains; (2) linker domain, consisting of N2a; and (3) CTD; consisting of N2b + N3 domains. The chimeric HiBiT-tagged N proteins were co-expressed with SARS-CoV2 membrane proteins, and secreted HiBiT levels were measured as reflections of VLP production and secretion (Figure 4B).

The results demonstrated a primary role for the nucleocapsid CTDs in VLP production and secretion (Figure 4B). This was most evident from results of two chimeras, designated N^MS^3 and N^SMS^5, both of which are ~80% as effective as complete SARS-2 N and both of which retain the SARS-2 CTDs (Figure 4B). However, there was also a minor role for the nucleocapsid NTDs, as evidenced by N chimeras with SARS-2 NTD or NTD + linker having ~40% the activity of complete SARS-2 N (Figure 4B). Linker domains did not contribute specifically to VLP production, as evidenced by the properties of N^MSM^6, which was equivalent to MERS N in support of VLPs. These results were independently confirmed by detecting VLP-associated S and M proteins in purified VLP preparations (Figure 4C, top VLP panels). Also of note, the diminished support of several VLP production processes were not due to reduced synthesis of chimeric N proteins, as measured by detection of viral proteins in cell lysates (Figure 4C, bottom panels). Overall, the findings demonstrate complex domain-specific contributions to VLP assembly that are largely dominated by the CTDs. These results are concordant with some previous reports demonstrating the role of coronavirus N-CTD in protein oligomerization and assembly [35,36].

### 3.4. A SARS-CoV2 Nucleocapsid Fragment Interferes with VLP Production

**Figure 5:**
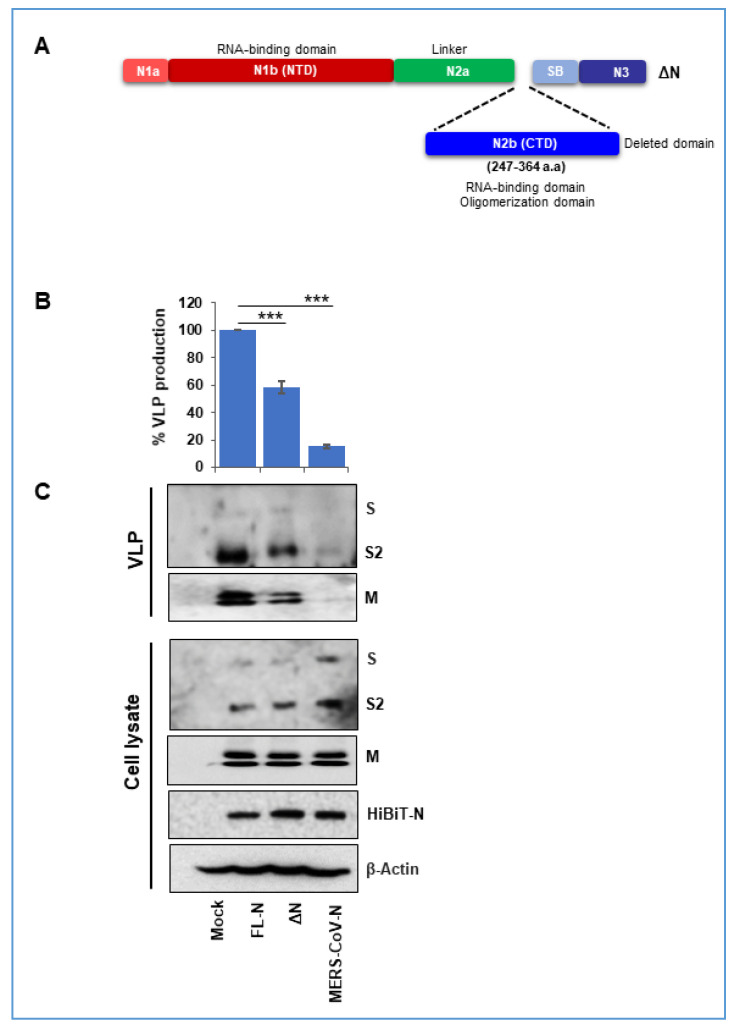
N proteins lacking CTDs interfere modestly with VLP production. (**A**) Schematic representation of CTD-deleted SARS-CoV2 N. (**B**) SARS-CoV2 N (FL-N) was cotransfected with S, E, and M plasmids, or in combination with CTD-deleted N, at 1:1 plasmid transfection ratio. The CTD-deleted N was not HiBiT-tagged. Yields of SEC-purified VLPs were determined by LgBiT complementation (NanoBiT technology. Results are means ± SE of three independent experiments. *** *p* < 0.001. (**C**) S, M, and N proteins in purified VLPs and cell lysates were detected by WB methods.

Further evaluation of specific nucleocapsid domains in VLP assembly were approached by asking whether SARS-2 nucleocapsid fragments might interfere with the natural VLP production process. To this end, a deleted form of N (ΔN) was constructed in which a portion of the CTD was eliminated (Figure 5). This ΔN construct, which lacks HiBiT, was co-transfected with the standard SARS-2 VLP assembly components, S, E, M, and HiBiT-N. The presence of ΔN significantly hindered in the assembly of VLPs and reduced VLP production by ~30% (Figure 5B), a marginal amount, consistent with the CTD of complete SARS-2 N protein having a dominant role in VLP assembly (Figure 4). Western blot detection of VLP proteins (Figure 5C) confirmed the finding that the ΔN fragment interfered weakly. These findings form the basis for continued tests of assembly interference by short nucleocapsid fragments or nucleocapsid peptidomimetics.

### 3.5. HiBiT-Tagged SARS-CoV2 VLPs Assess Virus Binding and Entry Events

**Figure 6:**
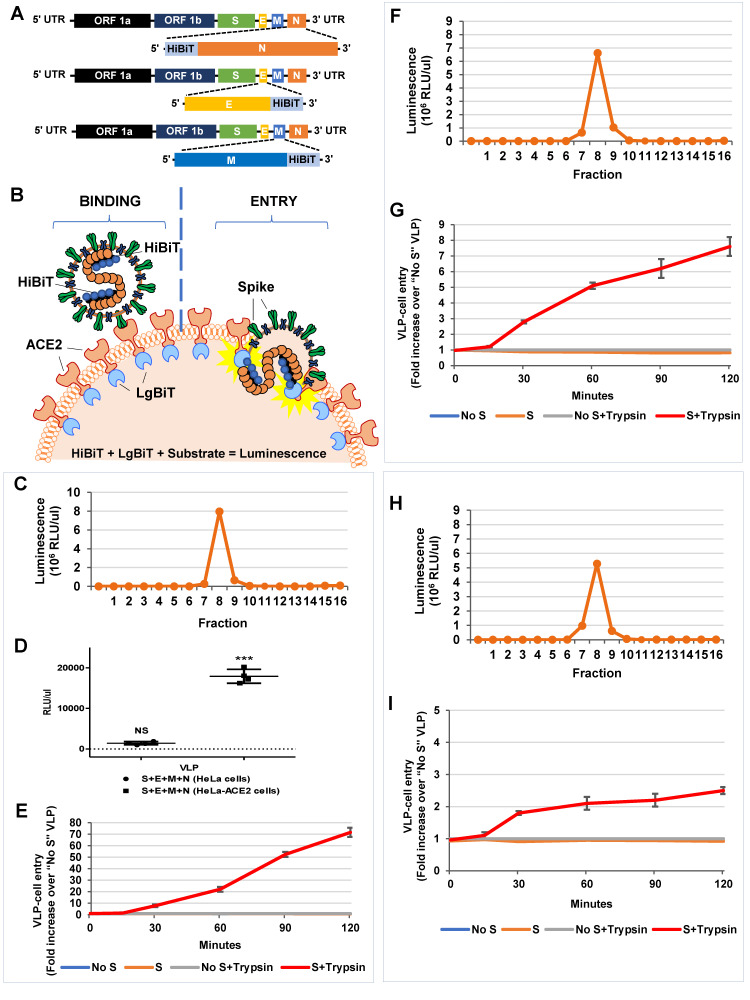
VLP binding and entry into target cells. (**A**) Schematic representation of the SARS-CoV2 HiBiT-N, E-HiBiT, and M-HiBiT constructs. HiBiT peptides were appended to the C-termini of E and M proteins. (**B**) Schematic depicting VLP binding (left) and entry (right) into target cells. VLPs bind to ACE2 receptors that have LgBiT appended to cytoplasmic C-termini. HiBiT-LgBiT complementation and development of Nluc measures successful VLP entry. (**C**) SEC elution profile depicting HiBiT-N VLPs in fractions 7–9. (**D**) VLP-cell binding; HeLa or HeLa-ACE2 cells were incubated with Nluc-N VLPs for 1.5 at 4 °C. VLPs were with or without S proteins; the VLPs lacking S served as controls. Cell-associated Nluc was measured by luminomoetry. Data are presented after subtraction of control binding of VLPs lacking S proteins (control VLP binding at y = 0). Results presented are means ± SE of four independent experiments. NS = non significant; *** *p* < 0.001. (**E**) VLP-cell entry; HeLa cells were transfected with ACE2-LgBiT plasmids. Twenty-four h later, HiBiT-VLPs, with or without S proteins, were inoculated at identical HiBiT input multiplicities, for 1 h at 4 °C. Vivazine (Nluc live cell substrate) and trypsin (20 ng/uL final concentration) were then added, with subsequent incubation at 37 °C (t = 0 min). Nluc levels were measured at the indicated time points. Results are presented as fold increase over signals generated by VLPs lacking S proteins. Data presented are means ± SD of three independent experiments. Significant Nluc accumulation over that generated by background “no S” VLPs was observed only when trypsin was present. (**F**,**H**) SEC elution profiles depicting E-HiBiT VLPs (**F**) and M-HiBiT VLPs (**H**) in fractions 7–9. (**G**,**I**) VLP-cell entry; E-HiBiT VLPs (**G**) and M-HiBiT VLPs (**I**) were evaluated for cell entry as described in (**E**).

CoV VLPs are tools to evaluate particle assembly (Figure 1, Figure 2, Figure 3, Figure 4 and Figure 5) and can also be used to assess particle entry into target cells. Indeed, HiBiT tagged flavivirus VLPs have been previously shown to be a useful quantitative method for the analysis of viral entry and release [37]. Here, we considered the potential of HiBiT-N VLPs in target-cell events, first in measuring virus-cell binding (Figure 6A). SEC-purified VLPs (Figure 6C) were applied to target cells in a 4 °C binding process. Definitive ACE2 receptor-dependent binding was observed (Figure 6D). Second, HiBiT-N VLPs were applied to target cells expressing a recombinant ACE2 in which Nluc “LgBiT” fragments were appended to ACE2 cytoplasmic termini. Here, VLPs were incubated at 37 °C, in the presence of S protein-activating trypsin proteases [38]. Nluc, assembled only after successful fusion of VLP and cell membranes, was measured at specific time points after 37 °C incubation. This was found to be a highly sensitive, accurate, and quantitative approach to measure SARS-CoV2 VLP entry, with signals up to 70-fold over background (Figure 6E). We quantified the efficiency of the HiBiT-N tagged VLP entry process by determining the maximal HiBiT-LgBiT complementation achieved after detergent-mediated lysis of virus-cell cultures, and by using this maximal value as a denominator. From these calculations, we estimated ~10% of VLPs successfully entered target cells in a 2 h incubation period.

We considered whether HiBiT tags could be placed on CoV structural proteins other than nucleocapsid, so that the membrane proteins might also be monitored using Nanobit approaches. To this end, we produced two more types of SARS-CoV2 VLPs having the HiBiT tags on C terminus of M and the E proteins. Production and SEC purification of the E- and M-HiBiT VLPs revealed that they could be obtained in yields similar to HiBiT-N VLPs (compare areas of HiBiT-containing fractions 7–9 in Figure 6C,F,H). In VLP-cell entry assays, the E-HiBiT and M-HiBiT VLPs were clearly effective at monitoring cell entry, however, they were ~10 and ~30-fold less robust than HiBiT-N VLPs in generating Nluc entry signals (compare Figure 6E,G,I). The relatively lower entry signals may be attributable to the limited mobilities of M and E proteins after VLP-cell fusion, and the resultant failure of many HiBiT moieties to reach LgBiT tags extending into cytosols from ACE2 transmembrane proteins. Nonetheless, these findings clearly demonstrate the potential for Nluc fragment-tagging and CoV VLP reagents in the study of CoV-cell entry processes.

## 4. Discussion and Conclusions

Here, we demonstrated that steps in SARS-CoV2 assembly and entry can be dissected using VLPs. We used the VLP system in two ways; first, to identify an underappreciated role for N proteins in SARS-CoV2 assembly, and second, to establish a platform for evaluating SARS-CoV2 cell entry with reductionist, quantitative assays that isolate the initial entry process from all other infection stages.

CoV N proteins carry out several functions in the context of natural infection. They suppress host cellular response to viral infection [39,40,41], and they arrest host cell cycling, thereby promoting viral replication [42,43]. N proteins interact with genomic RNA, and with virion E and M proteins, to integrate the coronavirus genome into virus particles [44]. Early reports focusing on CoV particle assembly did not reveal a role for N proteins [45], yet here in this study, N proteins were critical for SARS-CoV2 VLP production (Figure 3). This provided opportunities to dissect N domains for their roles in VLP assembly and secretion.

CoV N proteins are comprised of NTDs, central Ser/Arg (SR)-rich containing linker regions, and CTDs [24]. Our results demonstrated that SARS-CoV2 particle assembly and secretion was maximized by N proteins containing the SARS-CoV2 CTD (Figure 4). Conversely, N proteins lacking the CTD only modestly interfered with particle assembly (Figure 5), further implying that that the CTDs have a prominent role in assembly. The findings are consistent with a previous report suggesting a role for the CTD in N protein dimerization and oligomerization, both of which crucial in forming virus particles [36,46]. Other domains in addition to the CTD appeared to contribute more modestly to particle production. The linker (N2a) domain, when present with the CTD, moderately enhanced VLP production. This may arise because the SR-rich linker domain regulates N phosphorylation, oligomerization, and resulting higher order ribonucleoprotein assembly [47]. The strong CTD requirement could also come from the N3 domains that are present in CTD chimeras. N3 domains interact with M [48,49] at discontinuous regions of M endodomains [23,50,51], and may be central in promoting particle assembly.

A remaining question is whether N protein: RNA binding has a role in particle morphogenesis. Both NTDs and CTDs were associated with RNA binding in SARS-CoV [46,52], and N-RNA complexes may form prior to assembly into enveloped particles. At present, the VLP systems do not reveal the N: RNA interactions that take place in natural infections, and we have not yet investigated the relationships between N domain: RNA interactions and N domain functions in virus particle assembly.

The VLPs can, however, provide assay platforms for particle assembly and its inhibition. SARS-CoV2 assembly is a target for pharmacologic inhibition, and in silico approaches have identified several phytocompounds [53,54] and anti-viral and anti-microbial drugs [55,56], which may prove effective against SARS-CoV2. These computational methods help in designing of new drug candidates more rapidly, however, their actual validation in natural viral infection takes time and resources due to limitations of required biosafety facilities to handle highly pathogenic viruses. The VLP system provides a rapid and safe platform to validate computationally predicted antiviral compounds, and dissect antiviral operating mechanisms, as a step toward clinical application.

The VLP systems described here also provide platforms for evaluating virus-cell entry. Here, we found that the 11-amino acid HiBiT tags used to monitor VLP production and entry can be appended to different structural proteins of SARS-CoV2, as demonstrated by the VLP-cell entry assays (Figure 6). This advances the NanoBiT technology to SARS-CoV2, building from a recent report that has shown the utility of HiBiT tags on spike or membrane protein of infectious bronchitis coronavirus [57]. Using sensitive NanoBiT technology, the system isolates virus-cell entry and can thereby recognize the receptors and proteases operating as coronavirus-cell susceptibility factors (Figure 6). Notably, various sarbecovirus S proteins can be incorporated into VLPs, offering ways to determine whether newly-evolving SARS-CoV-2 variants of concern depend variably on host factors or exhibit distinctive cell entry pathways into target cells.

Overall, we report a HiBiT-VLP system that can be utilized in BSL-1 laboratory settings to quantify features of CoV assembly and entry. The VLPs were used here to illuminate roles for N protein domains in SARS-CoV-2 assembly and to demonstrate utility in virus-cell entry assays. We anticipate the VLP systems will be adaptable to screens for inhibitors targeting SARS-CoV2 entry and assembly, thereby accelerating antiviral drug discovery for COVID19 and other CoV diseases.
